# Water Distribution System Deficiencies and Gastrointestinal Illness: A Systematic Review and Meta-Analysis

**DOI:** 10.1289/ehp.1306912

**Published:** 2014-03-21

**Authors:** Ayse Ercumen, Joshua S. Gruber, John M. Colford

**Affiliations:** Division of Epidemiology, School of Public Health, University of California, Berkeley, Berkeley, California, USA

## Abstract

Background: Water distribution systems are vulnerable to performance deficiencies that can cause (re)contamination of treated water and plausibly lead to increased risk of gastrointestinal illness (GII) in consumers.

Objectives: It is well established that large system disruptions in piped water networks can cause GII outbreaks. We hypothesized that routine network problems can also contribute to background levels of waterborne illness and conducted a systematic review and meta-analysis to assess the impact of distribution system deficiencies on endemic GII.

Methods: We reviewed published studies that compared direct tap water consumption to consumption of tap water re-treated at the point of use (POU) and studies of specific system deficiencies such as breach of physical or hydraulic pipe integrity and lack of disinfectant residual.

Results: In settings with network malfunction, consumers of tap water versus POU-treated water had increased GII [incidence density ratio (IDR) = 1.34; 95% CI: 1.00, 1.79]. The subset of nonblinded studies showed a significant association between GII and tap water versus POU-treated water consumption (IDR = 1.52; 95% CI: 1.05, 2.20), but there was no association based on studies that blinded participants to their POU water treatment status (IDR = 0.98; 95% CI: 0.90, 1.08). Among studies focusing on specific network deficiencies, GII was associated with temporary water outages (relative risk = 3.26; 95% CI: 1.48, 7.19) as well as chronic outages in intermittently operated distribution systems (odds ratio = 1.61; 95% CI: 1.26, 2.07).

Conclusions: Tap water consumption is associated with GII in malfunctioning distribution networks. System deficiencies such as water outages also are associated with increased GII, suggesting a potential health risk for consumers served by piped water networks.

Citation: Ercumen A, Gruber JS, Colford JM Jr. 2014. Water distribution system deficiencies and gastrointestinal illness: a systematic review and meta-analysis. Environ Health Perspect 122:651–660; http://dx.doi.org/10.1289/ehp.1306912

## Introduction

Diarrheal diseases are responsible for a large health burden worldwide, causing approximately 10% of deaths among children < 5 years of age (Lozano et al. 2012). Diarrhea is also common in developed countries (Herikstad et al. 2002; Roy et al. 2006) and can have large economic implications in terms of medical expenditures and loss of workdays (Payment and Hunter 2001). One of the risk factors leading to this global disease burden is unsafe drinking water, both in developing and developed country settings (Black et al. 2003; Colford et al. 2006; Messner et al. 2006; Reynolds et al. 2008).

The focus of this review is drinking water–related diarrheal disease risk in settings where water is centrally distributed via a piped network. In such settings, diarrheal disease due to drinking water can be caused by contamination at the source, at the treatment plant (if any), in the distribution system, or at user end points (Craun et al. 2010). Here, we focus on (re)contamination of water in the distribution network before it reaches consumer taps, which can put consumers at risk of diarrheal illness even when the treatment plant effluent is in compliance with all drinking water quality regulations. Such contamination events are caused by deficiencies in the distribution system, including breach of physical pipe integrity (i.e., pipes can no longer provide adequate physical barrier against external contamination because of factors such as cross-connections with non-potable lines, fractures, leaky joints, or corrosion associated with aging), breach of hydraulic pipe integrity (i.e., pipes can no longer provide a reliable water supply in terms of volume or pressure due to factors such as main breaks, pump outages, or sudden changes in demand), and breach of water quality integrity (i.e., water quality deteriorates in pipes through factors such as decay of disinfectant residual) [National Research Council (NRC) 2006]. Both physical and hydraulic breaches are necessary for contamination to occur; lack of water pressure during hydraulic breaches allows external contamination to enter pipelines through the portals created by physical breaches. Entry of pathogens can be in the form of backflow from cross-connections or intrusion through leaks and cracks (Besner et al. 2011; LeChevallier et al. 2003). Aging water infrastructure in the United States and other developed countries makes water distribution systems particularly vulnerable to pathogen intrusion through increasingly frequent pipe breaks and other types of age-related deterioration as pipelines approach the end of their service lives [U.S. Environmental Protection Agency (EPA) 2011], and breaks, cracks and leaks in pipelines are also very common in the inadequately maintained and often overburdened water distribution systems of developing countries (Lee and Schwab 2005). The World Health Organization (WHO) recommends maintaining a chlorine residual of 0.2–0.5 mg/L in the distribution network to provide protection against pathogen intrusion in the event of breaches of physical and/or hydraulic pipe integrity (WHO 2011). However, not all networks maintain the recommended level of residual, and even in adequately chlorinated networks it has been debated whether the disinfectant residual can effectively inactivate intruding pathogens and preserve water quality integrity (Gadgil 1998; Payment 1999).

Links between waterborne disease outbreaks and distribution system deficiencies have been well documented in the United States and in developing countries (Craun and Calderon 2001; Craun et al. 2010; Lee and Schwab 2005). In contrast, the contribution of distribution systems to waterborne illness under non-outbreak conditions is not well understood. Risk assessment models have suggested distribution system problems as a potential risk factor for sporadic gastrointestinal illness (GII) (Lambertini et al. 2012; McInnis 2004; Teunis et al. 2010). Such models, however, typically rely on several assumptions. Findings from epidemiological studies on the association between distribution systems and endemic GII have been mixed; although previous reviews of limited scope on the subject exist (Colford et al. 2006; NRC 2006), the body of epidemiological evidence on endemic levels of GII due to distribution system deficiencies, to our knowledge, has not been systematically reviewed previously.

We conducted a systematic review and meta-analysis to investigate whether distribution system deficiencies are associated with increased risk of endemic waterborne illness in consumers of tap water. Our first research question was whether consumption of centrally treated and distributed tap water increases the risk of GII compared with consumption of tap water re-treated at the point of use (POU). By focusing on water that has been treated at a centralized facility and is safe to drink as it exits the treatment plant, we aimed to isolate the role of the distribution network from other potential causes of contamination at the source or treatment plant. Our second research question was whether reported distribution system problems, such as breach of physical, hydraulic, or water quality integrity in pipelines, is associated with increases in the risk of GII in tap water consumers served by piped networks.

## Methods

*Literature search*. We searched the Cochrane Library (http://www.thecochranelibrary.com/), PubMed (http://www.ncbi.nlm.nih.gov/pubmed), EMBASE (http://www.elsevier.com/online-tools/embase), and Web of Science (http://apps.webofknowledge.com/) for relevant published articles using combinations of the keywords “tap water, drinking water, distribution system(s), public water supply, OR municipal water supply” AND “diarrh(o)ea, diarrh(o)eal, gastrointestinal, gastroenteritis, OR gastritis.” We screened the titles and abstracts of articles for eligibility and reviewed full texts of relevant articles. In addition, the bibliographies of eligible articles were screened to identify additional studies.

*Selection criteria*. The primary inclusion criterion was that the measured exposure was consumption of tap water as obtained from the tap without further treatment. For studies comparing direct consumption of tap water with consumption of tap water re-treated at the POU, a subcriterion was that study participants received their tap water from centralized water treatment systems that did not report treatment failures at the time of the study and/or were reported to be in compliance with microbial water quality regulations. The second inclusion criterion was that the reported outcome was endemic GII under non-outbreak conditions, as reported by the authors. Multiple GII definitions were accepted, including diarrhea, gastroenteritis, acute gastrointestinal illness (AGI; defined as a combination of diarrhea and vomiting), highly credible gastrointestinal illness (HCGI; defined as different combinations of diarrhea, vomiting, nausea, and abdominal pain), highly credible gastroenteritis (HCG; definition similar to that of HCGI), and infections with specific diarrheagenic pathogens (e.g., *Campylobacter*). However, infections with protozoan pathogens such as *Cryptosporidium* and *Giardia* were excluded because these organisms can be resistant to water treatment (Steiner et al. 1997), making it difficult to isolate contamination occurring in the distribution system from treatment failure at the plant. The third inclusion criterion was the use of epidemiological methods to link exposures to health outcomes; studies using a risk assessment approach to infer GII outcomes from water quality data were excluded because they use theoretical transmission models that rely on several assumptions (Soller 2006) to estimate disease risk, in contrast to epidemiological methods that measure disease outcomes directly. Finally, because the objective of our review was to characterize the risk of endemic GII among general populations that are exposed to distribution system deficiencies, we excluded studies on specific subpopulations that are particularly vulnerable to GII from waterborne pathogens, such as the elderly and immunocompromised (Colford et al. 2005a, 2009; Gerba et al. 1996) or individuals that are not representative of a resident population, such as travelers (Ericsson 1998). The review was limited to studies in English, German, or Spanish (the languages spoken by the authors), with no limitations on study location or quality.

*Data extraction and meta-analysis*. Data were extracted independently by two unblinded authors (A.E. and J.S.G.), and discrepancies were resolved by discussion. Estimates of relative risk (RR), such as incidence density ratios (IDRs) and odds ratios (ORs), along with 95% confidence intervals (CIs) were extracted from the selected studies when available, and otherwise were calculated from the reported data using standard methods (Rothman et al. 2008). All relative measures were expressed such that a value > 1.0 indicates increased risk in the exposed group. If a study reported both unadjusted and adjusted estimates controlling for covariates, we used the adjusted estimates. If effect estimates for multiple age groups were reported, we extracted the estimates for all ages combined.

**Figure 1:**
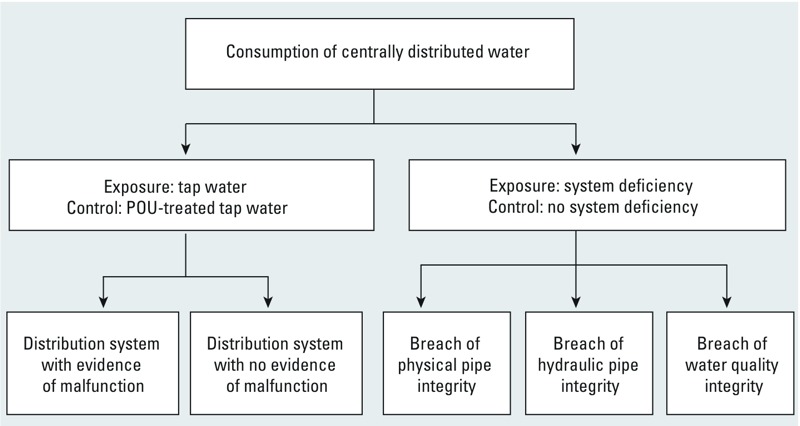
Categorization of studies for meta-analysis.

To address our research questions, we grouped the studies as follows: *a*) studies that compared consumption of tap water obtained directly from the tap with consumption of tap water re-treated at the POU, and *b*) studies that assessed the risk of GII associated with specific distribution system deficiencies. The second group was further subclassified according to previously defined categories of distribution system problems (NRC 2006) into studies that focused on *a*) breach of physical pipe integrity, such as cross-connections, cracks, and age-related pipe deterioration; *b*) breach of hydraulic pipe integrity, such as pressure loss in the network; and *c*) breach of water quality integrity, such as lack of adequate disinfection residual. We conducted a separate meta-analysis for each subgroup of studies (Figure 1) because we anticipated different types of distribution system deficiencies to have different health impacts, as well as different policy implications.

The meta-analysis was conducted using STATA (version 12; StataCorp., College Station, TX). We used fixed- and random-effects models with inverse variance weighting to pool the risk estimates, when appropriate. Heterogeneity was assessed using the Mantel-Haenszel chi-square statistic, and random-effects models were used when heterogeneity was detected (defined as a *p*-value < 0.20 on the chi-square statistic). Otherwise, fixed-effects models were used. Several factors were specified *a priori* as potential sources of heterogeneity, including location [developed vs. developing country according to the International Monetary Fund (IMF) definition of “advanced economies” vs. “emerging and developing economies” (IMF 2013)], characteristics of the study design (randomized vs. observational, blinded vs. nonblinded), and distribution system performance during the study period [continuously vs. intermittently operated, malfunctioning vs. nonmalfunctioning (Figure 1)]. For the purposes of our analysis, a malfunctioning system was defined *a priori* as one that had reported breaches of physical integrity (e.g., pipe breaks), breaches of hydraulic integrity (e.g., service intermittencies, low or negative pressure events), or breaches of water quality integrity (e.g., inadequate disinfectant residual in the network despite chlorination prior to distribution system entry). Subgroup analyses were performed to explore the effect of these factors on summary estimates. Sensitivity analyses were conducted to determine whether pooled estimates were disproportionately affected by any one study. We assessed publication bias using the Begg’s test, with a *p*-value < 0.20 interpreted as evidence for bias (Egger et al. 2008).

## Results

**Figure 2:**
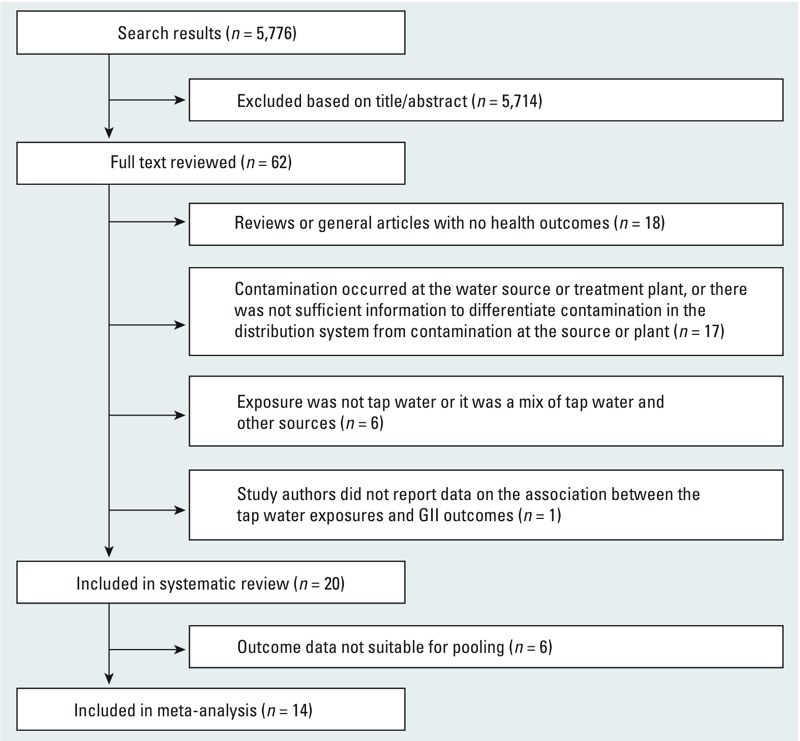
Flow chart showing inclusion and exclusion of articles. The number of search results reflects the omission of 469 duplicate citations from the total of 6,245.

Our original search resulted in a total of 6,245 studies, of which 469 were duplicates (i.e., repeat entries of citations from multiple databases). Thus, we screened titles and/or abstracts of 5,776 studies and reviewed the full text of 62 articles (Figure 2). The literature that we excluded based on title/abstract review included studies on waterborne disease outbreaks (including outbreaks caused by protozoan pathogens) and studies conducted in rural populations without access to a centralized water supply. Of the 62 articles we reviewed in full text, 20 studies were identified for inclusion in the systematic review, and 14 of these 20 studies had combinable data and were included in the meta-analysis. Ineligible studies reviewed in full text were excluded because *a*) they were reviews or general articles with no health outcomes (*n* = 18); *b*) contamination occurred prior to entry into the distribution system (at the water source or treatment plant), or there was not sufficient information to differentiate contamination in the distribution system from contamination at the source or plant (*n* = 17); *c*) exposure was not tap water or it was a mix of tap water and other sources (*n* = 6); or *d*) study authors did not report data on the association between the tap water exposures and GII outcomes (*n* = 1).

*Studies of tap water versus POU-treated tap water*. Six studies investigated the effects of consuming tap water versus POU-treated tap water (Table 1) (Colford et al. 2002, 2005b; Hellard et al. 2001; Payment et al. 1991, 1997; Semenza et al. 1998).

**Table 1:** Characteristics of studies of tap water versus tap water re‑treated at POU.

Study	Location	Source water	Treatment plant	Distribution system	Design	Comparison exposure	Effect estimate (95% CI)
Payment et al. 1991	Canada	River receiving sewage; coliforms and viruses detected	Conventional treatment with ozonation and chlorination; effluent in compliance with regulations; no coliforms or viruses in effluent	Negative pressures; inadequate residual	CRT, nonblinded	RO-treated water	IDR = 1.36 (1.10, 1.69)^*a*^
Payment et al. 1997	Canada	Same river as for Payment et al. 1991; coliforms, parasites, and viruses detected	Conventional treatment with ozonation and chlorination; effluent in compliance with regulations; no coliforms, parasites, or viruses in effluent	Same system as for Payment et al. 1991; no fecal coliforms; coliforms detected in 0.6% of samples	CRT, nonblinded	Ozonated bottles of RO-treated plant water or spring water	IDR = 1.14 (0.91, 1.42)^*a*^
Semenza et al. 1998	Uzbekistan	Not reported	Two-stage chlorination	Pressure-loss events; inadequate residual	Cohort,^*b*^ nonblinded	Chlorinated water	IDR = 2.61 (1.71, 4.00)^*a*^
Hellard et al. 2001	Australia	Protected forest catchments; fecal coliforms detected	Chlorination; no coliforms in effluent	Inadequate residual; no fecal coliforms; coliforms detected in 19% of samples	CRT, blinded	Microfiltration + UV	IDR = 1.00 (0.86, 1.15)
Colford et al. 2002	USA	River receiving agriculture and industry runoff; pathogens detected	Conventional treatment with chloramination; effluent in compliance with regulations	Not reported	CRT, blinded	Microfiltration + UV	IDR = 1.32 (0.75, 2.33)
Colford et al. 2005b	USA	River receiving sewage; parasites and viruses detected	Conventional treatment with chlorination; effluent in compliance with regulations; no coliforms, parasites, or viruses in effluent	No negative pressures; adequate residual; no coliforms	CRT, blinded	Microfiltration + UV	IDR = 0.96 (0.85, 1.08)
Abbreviations: CRT, cluster-randomized trial; IDR, incidence density ratio; POU, point of use; RO, reverse osmosis; UV, ultraviolet. ^***a***^Calculated from data reported in study. ^***b***^Observational arm within cluster-randomized trial.							

Study characteristics. Five of the studies were cluster-randomized trials (CRT), and one study was an observational analysis within a CRT (Table 1). In all studies, the exposed group consumed tap water directly from the tap without further treatment. In five studies, control group tap water was re-treated at the POU; one study provided households with bottles of treated plant water re-filtered by reverse osmosis or bottles of spring water, both of which were ozonated prior to bottling. Three studies achieved blinding by employing water treatment devices in the POU-treatment group that did not alter the taste of water and by providing households in the tap water group with a sham device that appeared to be identical to the active water treatment device. The remaining three studies were nonblinded. All six studies ascertained GII status through self-report.

Water system characteristics. Five of the studies were conducted in developed countries and one in a developing country (Table 1). The water system characteristics varied between the studies. The source water ranged from well-protected forest catchments to rivers heavily contaminated with sewage and runoff. Five studies provided source water quality data, and all five reported that pathogens or fecal indicator organisms were detected in the source water. The water treatment plants employed conventional treatment with chlorination or chloramination in four studies, and chlorination alone in two studies. Four studies reported the finished plant effluent to be in compliance with microbial water quality regulations, and none of the studies reported treatment plant failures during the study period. Four studies had a malfunctioning distribution system, as reported by study authors and by independent investigators (Besner et al. 2010; LeChevallier et al. 2002), one study was conducted in a system with no evidence of malfunctioning, and one study did not provide information on operation of the distribution system.

Summary of study findings. Four of the six studies shown in Table 1 reported positive associations between GII and consumption of tap versus POU-treated tap water, although associations reported by two of the studies were not statistically significant. The remaining two studies found no associations (Table 1). The Begg’s test suggested evidence of publication bias (*p* = 0.06). Significant heterogeneity was observed across the six studies (*p* < 0.0005); an overall pooled estimate was therefore not calculated.

**Figure 3:**
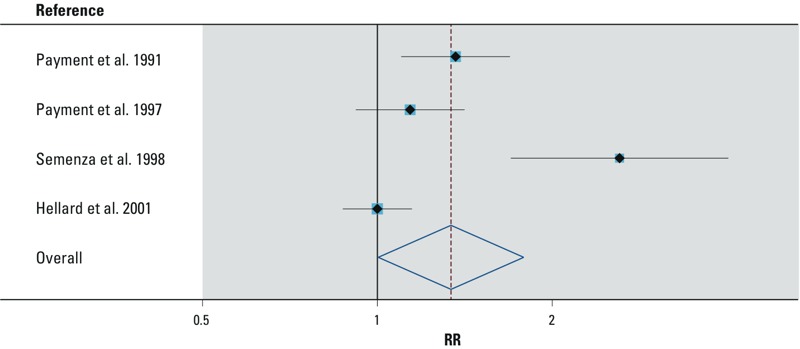
Random-effects meta-analysis of GII and tap water versus tap water re‑treated at point of use (POU) among studies in malfunctioning systems. Weights are from random-effects analysis. RR, relative risk. The pooled RR (95% CI) for these studies is 1.34 (1.00, 1.79). The measured exposure was consumption of centrally distributed tap water; participants received their tap water from centralized water treatment systems, and there was a documented malfunction in the distribution system.

We explored sources of heterogeneity by performing subgroup analyses with respect to study location (developed vs. developing country), study type (CRT vs. observational), blinding status (blinded vs. nonblinded), and distribution system performance during the study period (malfunctioning vs. nonmalfunctioning, based on reported data on network hydraulics and chlorine residual) (Table 2). The association reported by the one observational study conducted in a developing country (IDR = 2.61; 95% CI: 1.71, 4.00) was stronger than the random-effects pooled IDR based on the five randomized controlled trials in developed countries (IDR = 1.09; 95% CI: 0.95, 1.25; heterogeneity *p*-value = 0.05). Nonblinded studies in which participants in the intervention group were aware that they were consuming POU-treated tap water showed a significant increase in GII associated with direct tap water consumption (random-effects pooled IDR = 1.52; 95% CI: 1.05, 2.20); significant heterogeneity remained among these studies (*p* = 0.003). In contrast, there was no association based on the three blinded studies (fixed-effects pooled IDR = 0.98; 95% CI: 0.90, 1.08; heterogeneity *p*-value = 0.5). In the subset of studies with a malfunctioning distribution system, tap water was associated with a 34% increase in the rate of GII relative to the rate among treated-water consumers (random-effects pooled IDR = 1.34; 95% CI: 1.00, 1.79) (Figure 3) but significant heterogeneity remained (*p* < 0.0005). The association decreased when we excluded the developing country study (Semenza et al. 1998) that had more severe distribution system deficiencies, with approximately one-half of users reporting discernible pressure loss as opposed to transient low pressures recorded by loggers in the other studies (random-effects pooled IDR = 1.14; 95% CI: 0.95, 1.37; heterogeneity *p*-value = 0.06). Two of the studies in malfunctioning systems showed increasing risk of GII with increasing water consumption in the tap water group but not in the treated tap water group, by classifying the volume of water intake as a three-level categorical variable and conducting a trend test for the incidence of GII (Payment et al. 1991, 1997). The study that did not provide information on distribution system operation (Colford et al. 2002) reported a nonsignificant positive association between GII and consumption of tap water versus POU-treated tap water, and the study conducted in a properly operating system (Colford et al. 2005b) did not show an association.

**Table 2:** Meta-analysis of studies of tap water versus tap water re‑treated at POU.

Subgroup	No. of studies	IDR (95% CI)	Heterogeneity χ^2^ (*p*-value)^*a*^
Study type/location
CRT, developed country	5	1.09 (0.95, 1.25)	9.48 (0.050)
Cohort, developing country	1	2.61 (1.71, 4.00)	NA
Blinding
Nonblinded	3	1.52 (1.05, 2.20)	11.40 (0.003)
Blinded	3	0.98 (0.90, 1.08)	1.25 (0.534)
Distribution system
Malfunctioning system	4	1.34 (1.00, 1.79)	20.28 (< 0.0005)
Nonmalfunctioning system	1	0.96 (0.85, 1.08)	NA
No data on system	1	1.32 (0.75, 2.33)	NA
Abbreviations: CRT, cluster-randomized trial; IDR, incidence density ratio; NA, not applicable (only one study in subgroup); POU, point of use. ^***a***^χ^2^ Statistic with a *p*-value < 0.20 defined as evidence of heterogeneity.			

*Studies on loss of physical pipe integrity*. Six studies focused on loss of physical pipe integrity (Table 3) (Abu Amr and Yassin 2008; D’Argenio et al. 1995; Mohanty et al. 2002; Nygård et al. 2004; Tinker et al. 2009; Yassin et al. 2006).

**Table 3:** Characteristics of studies that focused on physical pipe integrity.^*a*^

Study	Location	Source water	Treatment plant	Distribution system	Design	Exposure	Comparison exposure	Outcome assessment	Effect estimate (95% CI)
D’Argenio et al. 1995	Italy	Not reported	Not reported	Presumed to be continuously operated; some chlorine residual; total coliforms, fecal coliforms, and fecal streptococci detected in affected pipe segment	Cohort	Pipeline with fecal contamination from cross-connections	Pipeline with no fecal contamination	Self-report	RR = 2.67 (1.16, 6.11)^*b*^
Mohanty et al. 2002	India	Surface water	Conventional treatment with chlorination	Intermittently operated; inadequate residual; total and fecal coliforms detected	Ecological	Unit increase in percentage of cast-iron pipes in service zone	NA	Self-report	Regression coefficient, –0.42 (*p *= 0.10)
Yassin et al. 2006	Palestine	Groundwater	Chlorination	Intermittently operated; inadequate residual; fecal contamination detected more often than at the source	Cross-sectional	Network > 1 year old	Network ≤ 1 year old	Self-report	RR = 1.51 (0.80, 2.83)^*b*^
Abu Amr and Yassin 2008	Palestine	Groundwater	Chlorination	Intermittently operated; inadequate residual; fecal contamination detected more often than at the source	Cross-sectional	Network > 1 year old	Network ≤ 1 year old	Self-report	RR = 1.03 (0.68, 1.56)^*b*^
Nygård et al. 2004	Sweden	Surface and groundwater	Chlorination (for surface water only)	Presumed to be continuously operated; low-level residual	Ecological	10-m increase in pipe length per person in municipality	NA	Surveillance records	IDR = 1.12 (1.08, 1.16)
Tinker et al. 2009	USA	Not reported	Not reported	Presumed to be continuously operated; adequate residual	Ecological	ZIP code with long hydraulic residence time	ZIP code with intermediate hydraulic residence time	Emergency department records	OR = 1.06 (1.04, 1.08)^*c*^
Abbreviations: IDR, incidence density ratio; NA, not applicable; OR, odds ratio; RR, relative risk. ^***a***^Results not pooled. ^***b***^Calculated from data reported in the study. ^***c***^Pooled from estimates for two utilities reported in the study.									

Study characteristics. One of the studies presented in Table 3 assessed the impact of cross-connections with sewer lines (D’Argenio et al. 1995), one used pipe material as a proxy for physical integrity (Mohanty et al. 2002), two focused on pipe age as a proxy for age-related deterioration (Abu Amr and Yassin 2008; Yassin et al. 2006), and two focused on pipe length and hydraulic residence time as an aggregate measure (Nygård et al. 2004; Tinker et al. 2009) (longer pipelines are more likely to have a larger number of leaks and fractures, and there are more opportunities for intrusion of pathogens through these when the water remains in pipes longer). Data on pipe characteristics were obtained from water utilities or reported by participants. With the exception of one study during which there was media awareness about fecal contamination in the network as a result of the cross-connections (D’Argenio et al. 1995), participants were effectively blinded to their exposure status because knowledge of the physical state of water pipes as a risk factor for GII was presumably limited in study populations. GII outcomes were assessed by surveillance records or from self-reported symptoms.

Water system characteristics. Three of the studies were conducted in developed countries and three in developing countries (Table 3). The developed countries presumably had continuously operated distribution systems, whereas the operation of the studied distribution systems in the three developing countries was reported to be intermittent, with water delivered for a limited number of hours per day. All studies were conducted in chlorinated systems. D’Argenio et al. (1995) did not provide information on the level of the chlorine residual in the distribution network; the rest were conducted in chlorinated systems with varying levels of residual.

Summary of study findings. The studies shown in Table 3 showed a range of positive associations between GII and the different exposures related to loss of physical pipe integrity, although some of the effect estimates were close to the null and three of the studies reported associations that were not statistically significant. Because of the differences in the exposure definitions among the studies, we did not conduct a meta-analysis on health outcomes; instead we summarized the general findings of the individual studies. Residing on a street served by a pipeline with fecal contamination due to cross-connections between water and sewer lines was associated with the occurrence of self-reported GII symptoms in tap water consumers compared with residing on a socioeconomically similar nearby street served by an uncontaminated pipeline (D’Argenio et al. 1995). The unit increase in the percentage of cast-iron water pipes, instead of more leak-prone materials, in a given service area appeared protective against self-reported GII outcomes aggregated at the service area level (Mohanty et al. 2002). In two studies (Abu Amr and Yassin 2008; Yassin et al. 2006), increased illness was observed in consumers served by networks > 1 year old (compared with networks ≤ 1 year old), but the effect estimate was very close to the null in one of these studies (Abu Amr and Yassin 2008), and both studies had low precision. Two studies reported positive associations of GII with increasing water residence time in the distribution system. One of these studies reported that the incidence of *Campylobacter* infections increased with every 10-m increase in water pipe length per person in a given service area (defined as the total length of the distribution network in the municipality divided by the number of people served) (Nygård et al. 2004). The other study (Tinker et al. 2009) investigated the association between GII and hydraulic residence time (i.e., time it takes for water to travel from the treatment plant to consumer taps), which was calculated from the water utilities’ hydraulic models, aggregated by ZIP code and defined as a categorical variable with three levels (short, intermediate, long) based on the 10th and 90th percentiles. The authors reported that people living in ZIP codes with long water residence time were slightly more likely to have medical visits related to GII than those living in ZIP codes with intermediate water residence time, but there was no difference in GII between people from ZIP codes with intermediate versus short residence times (Tinker et al. 2009). In addition, two of the previously discussed CRTs (Payment et al. 1991, 1997) had mixed findings on the impact of distance from the treatment plant on GII. Secondary analysis of the data from Payment et al. (1991) showed that increasing distance from the treatment plant (classified as a five-level categorical variable) showed increasing IDR for GII for consumption of tap water versus POU-treated tap water (Payment et al. 1993), whereas Payment et al. (1997) reported no association between GII and hydraulic residence time (method of analysis not specified by authors).

*Studies on loss of hydraulic pipe integrity.* Nine studies investigated the effects of loss of hydraulic pipe integrity (Table 4) (Abu Amr and Yassin 2008; Abu Mourad 2004; Cifuentes et al. 2002; Fewtrell et al. 1997; Huang et al. 2011; Hunter et al. 2005; Nygård et al. 2007; Özkan et al. 2007; Yassin et al. 2006).

**Table 4:** Characteristics of studies of hydraulic pipe integrity.^*a*^

Study	Location	Source water	Treatment plant	Distribution system	Design	Exposure	Comparison exposure	Outcome assessment	Effect estimate(95% CI)
Continuous systems
Fewtrell et al. 1997	United Kingdom	Not reported	Not reported	Not reported	Ecological	No. of water outages in a ZIP code area	—	Surveillance records	Correlation coefficient: Shigella 0.42 (*p *= 0.07)^*a*^^,^^*b*^^**^Hep A 0.67 (*p *= 0.001)^*a*^^,^^*b*^
Hunter et al. 2005	United Kingdom	Not reported	Effluent in compliance with regulations	Not reported	Cross-sectional^*c*^	Water outage	No water outage	Self-report	OR = 12.50 (3.49, 44.71)
Nygård et al. 2007	Norway	Not reported	Not reported	Not reported	Cohort	Water outage	No water outage	Self-report	OR = 2.00 (1.30, 3.20)
Nygård et al. 2007	Norway	Not reported	Not reported	Not reported	Cross-sectional^*d*^	Water outage > 6 hr duration	Water outage ≤ 6 hr duration	Self-report	OR = 1.90 (1.00, 3.40)^*b*^
Özkan et al. 2007	Turkey	Not reported	Not reported	Not reported	Cross-sectional	Water outage > 12 hr duration	No water outage > 12 hr duration^*e*^	Self-report	OR = 10.28 (2.95, 35.48)
Huang et al. 2011	Taiwan	Not reported	Not reported	Not reported	Ecological	Days with water outage^*f*^	10 days with normal water supply before water outage	Hospital records	IDR = 1.31 (1.26, 1.37)
Intermittent systems
Cifuentes et al. 2002	Mexico	Groundwater	Chlorination	Not reported	Cross-sectional	Intermittent supply	Full-day supply	Self-report	OR = 2.00 (1.16, 3.70)
Abu Mourad 2004	Palestine	Groundwater	Not reported	Not reported	Cross-sectional	Intermittent supply	Full-day supply	Self-report	OR = 1.53 (1.15, 2.03)^*g*^
Yassin et al. 2006	Palestine	Groundwater	Chlorination	Inadequate residual	Cross-sectional	Intermittency of > 1 day duration	Intermittency of 1-day duration	Self-report	RR = 1.33 (0.92, 1.91)^*g*^
Abu Amr and Yassin 2008	Palestine	Groundwater	Chlorination	Inadequate residual	Cross-sectional	Intermittency of > 1 day duration	Intermittency of 1-day duration	Self-report	RR = 1.49 (1.06, 2.09)^*g*^
Abbreviations: IDR, incidence density ratio; OR, odds ratio; RR, relative risk.^***a***^Results from 1991; no summary result reported by authors for all years in study; authors found no evidence of a causative temporal link at individual level. ^***b***^Not included in pooled analyses. ^***c***^Cross-sectional analysis within control group of case–control study. ^***d***^Cross-sectional analysis within exposed group of same cohort study. ^***e***^Authors specified water outages > 12 hr as a binary exposure variable; we assumed that the comparison exposure includes outages ≤ 12 hr as well as no outages. ^***f***^Effect estimate was similar when the 10-day period following the outage was used as the exposure. ^***g***^Calculated from data reported in study.									

Study characteristics. The exposure in five studies was temporary pressure loss at the tap (i.e., water outage) typically caused by main breaks or repair work in otherwise continuously operated distribution networks (compared with no water outages), and in four studies the exposure was chronic outages associated with intermittent water delivery (compared with uninterrupted full-day delivery) or the duration of such chronic outages (> 1 day vs. 1 day) (Table 4). The studies obtained water outage data from water utilities or through self-report by participants. By the nature of the exposure, participants were nonblinded to their exposure status as loss of pressure at the tap was evident to study participants; however, knowledge of pressure loss as a risk factor for GII was presumably limited. GII symptoms were ascertained from surveillance or hospital data or from self-reported symptoms.

Water system characteristics. Of the five studies of continuous distribution systems, all but one were conducted in developed countries, whereas the four studies of intermittent systems were all conducted in developing countries (Table 4). None of the studies of continuous systems provided additional information on water system characteristics, with the exception of one study that reported that the water utility was compliant with drinking water regulations (Table 4). Among the studies of intermittent systems, one did not specify whether the source water was chlorinated before distribution. The other three were conducted in chlorinated networks and, of these, only two reported the level of residual.

**Figure 4:**
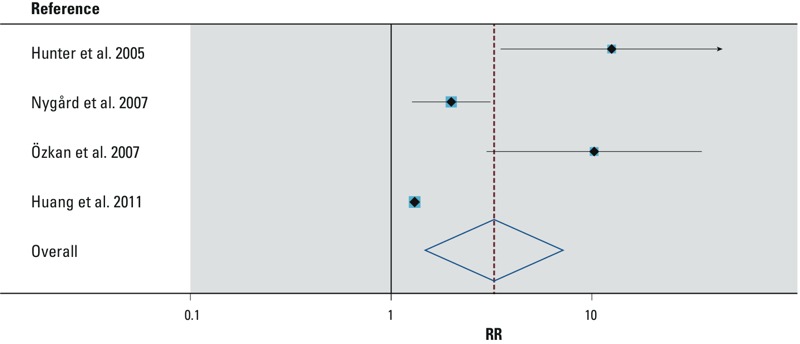
Random-effects meta-analysis of GII and water outage in continuous systems. The measured exposure was consumption of centrally distributed tap water; participants experienced a breach of hydraulic pipe integrity through water outages in otherwise continuously operating distribution systems. RR, relative risk. Weights are from random-effects analysis. The pooled RR (95% CI) for these studies is 3.26 (1.48, 7.19).

Summary of study findings. All nine of these studies suggested an increased risk of GII associated with water outages, in both continuously and intermittently operated systems (Table 4). Because of inherent operational differences between intermittent and continuous distribution networks, studies in these categories were analyzed separately. Among the five studies in continuous systems, one study (Fewtrell et al. 1997) was excluded from the pooled analysis because it only reported a correlation coefficient (but did not find evidence for a causative temporal link). For the remaining four studies, the Begg’s test suggested evidence of publication bias (*p* = 0.04). The pooled analysis showed a significant increase in GII associated with water outages compared with normal operation without outages (random-effects pooled RR = 3.26; 95% CI: 1.48, 7.19) (Figure 4) with significant heterogeneity among studies (*p* < 0.0005). Limiting the analysis to studies in developed countries (i.e., excluding Özkan et al. 2007) somewhat reduced the pooled estimate (random-effects pooled RR = 2.34; 95% CI: 1.13, 4.86) but did not eliminate the heterogeneity (*p* < 0.0005). Nygård et al. (2007) reported increased GII when the outages lasted > 6 hr versus < 6 hr (OR = 1.90; 95% CI: 1.00, 3.40) in the group that had outages. They also reported increased GII with increasing water consumption (> 1 vs. ≤ 1 glass per person per day) in the study group that experienced outages but not in the unexposed group.

For studies in intermittently operated systems, we could not assess publication bias because of the small number of studies in each exposure category. The pooled analysis of the two studies on chronic intermittencies in water delivery showed increased odds of GII (fixed-effects pooled OR = 1.61; 95% CI: 1.26, 2.07; heterogeneity *p*-value = 0.4) compared with full-day water supply. The pooled analysis of the two studies on the duration of intermittencies showed a significant increase in GII with intermittencies lasting longer than 1 day (fixed-effects pooled RR = 1.42; 95% CI: 1.11, 1.82; heterogeneity *p*-value = 0.7) compared with intermittencies lasting 1 day.

*Studies on loss of water quality integrity*. Three studies assessed the effects of low or nondetectable residual in the distribution system despite centralized chlorination prior to distribution (Table 5). (Egorov et al. 2002; Mohanty et al. 2002; Semenza et al. 1998).

**Table 5:** Characteristics of studies of water quality integrity.^*a*^

Study	Design	Exposure	Comparison exposure	Outcome assessment	Effect estimate (95% CI)
Semenza et al. 1998	Cross-sectional^*b*^	Nondetectable chlorine in household water sample from piped supply	Detectable chlorine in household water sample from piped supply	Self-report	IDR = 1.60 (0.70, 3.70)
Egorov et al. 2002	Cross-sectional	Interquartile range (0.22 mg/L) decrease in chlorine	NA	Self-report	IDR = 1.42 (1.05, 1.91)
Mohanty et al. 2002	Ecological	Unit increase in percentage of distribution system samples with nondetectable chlorine in service zone	NA	Self-report	Regression coefficient 0.46 (*p *= 0.64)
Abbreviations: IDR, incidence density ratio; NA, not applicable. ^***a***^Results not pooled. ^***b***^Cross-sectional analysis within exposed group of cluster-randomized trial.					

Study characteristics. The exposure definitions varied among the studies (Table 5). Semenza et al. (1998) focused on lack of detectable chlorine residual at the tap compared to detectable residual; Egorov et al. (2002) estimated the effect of an interquartile (0.22 mg/L) decrease in free chlorine residual in the network (relative to the residual in the plant effluent); and Mohanty et al. (2002) focused on the effect of a unit increase in the percentage of distribution system samples without detectable residual within a given service area. Exposure was assessed by measurement of chlorine residual by the utility or study investigators, and GII outcomes were ascertained through self-report in all three studies.

Water system characteristics. Two studies (Mohanty et al. 2002; Semenza et al. 1998) were conducted in previously described distribution systems with intermittencies in delivery (Tables 1 and 3), and one (Egorov et al. 2002) was conducted in a system serving conventionally treated and chlorinated groundwater via a network with variable water pressure in different parts but no reported pressure loss events.

Summary of study findings. All three of these studies suggested an association between GII and lack or decrease of chlorine residual, but only one study had a statistically significant effect estimate (Table 5). Egorov et al. (2002) noted a correlation between decreasing chlorine residual and increasing distance from the plant, suggesting residence time in the network as a potential causal factor behind the association between the decrease in chlorine residual and increase in GII. Because of the differences in study designs and exposure definitions among the studies, a meta-analysis was not performed.

## Discussion

Our review of studies comparing consumption of tap water with that of POU-treated water suggests that directly consuming tap water is associated with GII outcomes in settings where distribution systems are documented to have deficiencies, such as low-pressure events or inadequate disinfectant residual (IDR = 1.34; 95% CI: 1.00, 1.79) (Table 2). No significant association was observed in the only study carried out in a properly functioning distribution system (Colford et al. 2005b). A pooled estimate based on the subset of three nonblinded studies indicated a significant positive association between GII and consumption of tap water versus POU-treated water (IDR = 1.52; 95% CI: 1.05, 2.20); however, there was no association based on the three blinded studies (IDR = 0.98; 95% CI: 0.90, 1.08) (Table 2). In our review, we also identified articles that focused on specific system deficiencies. We found that GII was significantly associated with water outages in continuously operated distribution systems (RR = 3.26; 95% CI: 1.48, 7.19) and with chronic outages in intermittent systems (OR = 1.61; 95% CI: 1.26, 2.07). In both types of systems, longer outages were associated with increased risk of GII compared with shorter outages. Other network deficiencies, such as breach of physical pipe integrity or lack of chlorine residual, were also associated with GII outcomes. Taken together, these findings suggest that (re)contamination of drinking water in distribution systems can present a health risk for consumers served by piped water networks.

Our findings indicate evidence of publication bias, suggesting that studies with positive findings may have been preferentially published over those with null or inconclusive findings. It is therefore possible that our pooled effect estimates are higher than the true health risk associated with distribution systems. Moreover, our review indicates that there are relatively few studies to date that focus on this critical topic, suggesting the need for further research.

*Heterogeneity between study settings and designs*. There was significant heterogeneity among study settings and water system characteristics. We used meta-analysis as a tool to explore the effect of these heterogeneities on study findings. Studies conducted in similar settings were combined, and pooled estimates were contrasted between such subgroups to highlight important differences (e.g., between continuous and intermittent systems or malfunctioning and nonmalfunctioning networks). However, significant heterogeneity often remained even within subgroups.

One potential source of remaining heterogeneity, even after classifying studies as those conducted in malfunctioning versus nonmalfunctioning systems, is that myriad factors can influence the health risk associated with distribution systems, such as the number and size of leaks and cracks in pipes, the levels of fecal contamination present in the vicinity of pipelines, the magnitude and frequency of pressure loss events, and the levels of disinfectant residual in the affected pipe segments (LeChevallier et al. 2003). Broadly classifying networks as malfunctioning versus nonmalfunctioning based on system-wide performance data provides a basic tool for comparison, but given the expected temporal and spatial variability in these factors, it is not surprising that our classifications did not fully capture the heterogeneity across studies. Moreover, most distribution systems have cracks and leaks as evidenced by water losses, which can be as high as 32% in U.S. utilities (LeChevallier et al. 2003) and > 40% in developing countries (Lee and Schwab 2005), suggesting that no distribution system is truly nonmalfunctioning. However, the presence of cracks and leaks alone is not sufficient for pathogen intrusion, given that the network maintains adequate pressure and disinfectant residual (Besner et al. 2011). This suggests that our classification of networks with adequate levels of residual and no documented pressure loss during the study as “nonmalfunctioning” is consistent with the principles of pathogen intrusion into pipes and that our findings are relevant to the contexts under which most systems operate. Nonetheless, collection of more fine-grained data on these system parameters could improve the interpretation of future studies.

Study designs varied widely among articles included in our review. Although the studies comparing consumption of tap water versus POU-treated water almost exclusively employed randomized designs, studies of specific distribution system characteristics used observational methods including cohort, cross-sectional, and ecological designs. Observational studies varied in their attempts to control for confounding; some reported unadjusted estimates whereas others controlled for confounding. Factors that investigators controlled for (e.g., household income, hygiene practices, sanitation and sewerage facilities) were not consistent across studies. The most common observational design was cross-sectional studies. One potential flaw of this design is the inability to establish temporality (Rothman et al. 2008). Ecological studies were also commonly used to study network characteristics at service area levels, and this design is vulnerable to the “ecological fallacy” in which associations observed between aggregate exposures and outcomes may not reflect true causal relationships at the individual level (Piantadosi et al. 1988). Regardless, in our review we found that results were generally consistent (effect measures > 1 associated with distribution system deficiencies), despite the differences in study designs.

*Potential limitations of studies*. Recall bias. In studies with self-reported outcomes (e.g., diarrhea symptoms), there is evidence from the literature that exposure status can influence symptom recall and consequently effect measures (Colford et al. 2009; Hunter 2009; Schmidt and Cairncross 2009). Consistent with this evidence, the subset of nonblinded studies in our review showed a significant association between self-reported GII and consumption of tap water versus POU-treated water, yet there was no evidence of an association in blinded studies. We could not explore the role of lack of blinding separately from malfunctioning systems because of overlap between studies; therefore, we cannot rule out recall bias. Studies in our review that focused on specific network deficiencies such as water outages were nonblinded by the nature of the exposures studied. One of these studies assessed the impact of participants’ knowledge of their exposure status on the findings (Nygård et al. 2007). In that study, investigators selected participants who experienced an outage based on water utility data. The investigators then asked participants whether they thought there was a main break or repair in the pipes supplying their homes; 75% of exposed participants replied “yes” compared with 25% of unexposed participants (*p* < 0.001), indicating awareness of exposure. However, stratified analyses among participants who believed they were exposed versus unexposed showed similar associations between water outage and GII, suggesting that any bias in reporting of outcomes due to lack of blinding had a negligible impact on their findings. In another study assessing the impact of cross-connections, D’Argenio et al. (1995) reported increased recall of GII symptoms during the period when there was media awareness about fecal contamination in the pipes. The authors found higher rates of GII in both exposed and unexposed groups during this period compared with when the presence of contamination was not yet publicly known. However, the exposed group with cross-connections had higher risk of GII than the unexposed group during both periods, again suggesting that the findings are robust to recall bias. Nonetheless, objective measures of waterborne illness, such as pathogen-specific antigen responses, could improve future reporting of nonblinded studies evaluating the impact of water distribution systems on the health of consumers. Negative control outcomes (i.e., outcomes that are not expected to be affected by tap water exposure) can be used to assess the magnitude of recall bias when measuring objective outcomes is not feasible (Lipsitch et al. 2010).

Water contamination prior to distribution system entry. In studies comparing tap water with POU-treated water, it is possible that water contamination prior to distribution—rather than within the distribution system—is responsible for the increase in GII in tap water consumers. In the studies we reviewed, plant effluent was in compliance with regulations and no treatment failures were reported. However, regulatory standards are often based on indicator organisms for fecal contamination, whose ability to predict disease is controversial (Gundry et al. 2004). Only three studies performed additional tests for selected human enteric viruses and parasites in the finished plant water, and no such organisms were detected (Colford et al. 2005b, as reported by LeChevallier et al. 2002; Payment et al. 1991, 1997). For the remaining studies, one cannot exclude the possibility that the plant effluent may contain disinfection-resistant pathogens such as *Cryptosporidium*, whose absence cannot be confirmed by the absence of indicator organisms (Gadgil 1998). Short-term imperfections in plant performance (e.g., transient breakthrough of turbidity from filters) and low-level or sporadic breakthrough of pathogens have also been suggested as mechanisms for contamination that would not be detected by routine plant performance monitoring (Payment and Hunter 2001). One study in our review did have an additional study arm consuming finished plant water that had been bottled before distribution system entry (Payment et al. 1997). GII in that group was similar to the group consuming POU-treated water and significantly lower than the tap water group. Although we cannot rule out the role of water contamination prior to distribution for the other studies in our review, Payment et al. (1997) were able to isolate the distribution system as the source of contamination. Similar methods could improve the interpretation of future studies.

Water contamination at user end points. One limitation of the studies that investigated the effects of water outages is their failure to account for water handling and hygiene practices during the outages. In these studies, the observed increase in illness may have been mediated by altered practices in the household during the intermittencies in service, as opposed to pathogen intrusion into pipelines during pressure loss in the system. Such practices could include reverting to alternate sources of water that are of poor quality, secondary contamination of stored water in the household (Mintz et al. 1995), or poor hygiene due to reduced quantities of available water (Esrey et al. 1991). One study in our review (Huang et al. 2011) reported no deterioration in tap water quality following water outages and also found increases in skin and eye infections associated with outages, suggesting that an alternative water-washed pathway was the primary risk factor for the observed increase in GII symptoms. Their results would not change the general conclusion that service disruptions are associated with an increased risk of GII, but they would have different policy implications, with emphasis on preventing water outages instead of establishing measures to minimize pathogen intrusion during an outage. However, in another study included in our review, Nygård et al. (2007) reported that the odds of GII were reduced when the pipe segment affected by the main break or repair work was flushed or rechlorinated, suggesting that pathogen intrusion into pipelines during periods of pressure loss is at least partly responsible for increased illness.

*Dose–response relationships*. Findings from several studies in our review suggested increasing GII with increases in volume of tap water consumed, duration of a water outage, and residence time of water in the distribution system. Volume of contaminated tap water intake would be expected to predict consumers’ ingested pathogen load. Payment et al. (1991, 1997) classified the volume of water intake as a three-level categorical variable and detected a significant trend in GII incidence with respect to these categories, whereas Nygård et al. (2007) showed higher risk of GII in households consuming > 1 versus ≤ 1 glass of water per person per day. Importantly, a positive dose–response relation with water consumption was reported only in tap water consumers in malfunctioning systems; participants with POU-treated water (Payment et al. 1991, 1997) or those not exposed to water outages (Nygård et al. 2007) did not show evidence of increasing GII with increasing water consumption. Along similar lines, increasing duration of water outages would make pipes vulnerable to backflow and intrusion for longer periods. Three studies showed increased GII associated with longer versus shorter outages (Abu Amr and Yassin 2008; Nygård et al. 2007; Yassin et al. 2006). Finally, longer pipelines would have a larger number of cracks and leaks, increasing the number of potential portals for pathogen intrusion. One study (Nygård et al. 2004) showed increasing GII per 10-m increase in pipe length per person, whereas Tinker et al. (2009) found that ZIP codes with long hydraulic residence time in the distribution system were at higher risk of GII than ZIP codes with intermediate residence time. Analysis of data from Payment et al. (1991) showed increasing IDR for tap water consumption versus POU-treated water consumption in zones more distant from the treatment plant (Payment et al. 1993). Increasing volume of tap water consumption, duration of water outages, and hydraulic residence time would all be expected to increase opportunities for ingestion of water recontaminated in distribution pipes and elevate the risk of GII, which is consistent with the findings of our review.

## Conclusions

Although it is well established that large disruptions in water distribution systems can cause outbreaks of waterborne illness (Craun and Calderon 2001; Craun et al. 2010; Lee and Schwab 2005), we believe this review to be the first systematic review of the available published evidence of the impact of routine distribution system problems on low-level, background GII. The evidence we present here suggests that tap water consumption is associated with endemic GII in malfunctioning distribution networks. Specific system deficiencies, such as loss of pipe integrity, water outages, and inadequate residual, are also associated with increased risk of GII. Although the available evidence does not allow us to rule out noncausal mechanisms for this association, the consistency of our findings justify further research on this critical topic.

Randomized controlled trials comparing tap water consumption to consumption of POU-treated water are a strong study design for characterizing health risk from overall deficiencies of distribution systems. Prospective cohort studies that use utility data to identify system failures and follow-up with affected and unaffected tap water consumers follow a study design suitable for investigating the health impact of specific distribution system problems and allow establishing temporality between exposures and outcomes. Future studies should, ideally, include *a*) blinding or objective outcomes to minimize recall bias, *b*) collect more detailed water system measurements relevant to the homes of participants to better characterize individual exposure to distribution system problems, and *c*) measure microbiological water quality at key points between the water treatment plant and the point of consumption to differentiate contamination occurring in the distribution system from treatment plant failures or POU contamination.
